# Is the Comparison between Exercise and Pharmacologic Treatment of Depression in the Clinical Practice Guideline of the American College of Physicians Evidence-Based?

**DOI:** 10.3389/fphar.2017.00257

**Published:** 2017-05-15

**Authors:** Yael Netz

**Affiliations:** Behavioral Studies, Graduate School, The Academic College at WingateWingate, Israel

**Keywords:** antidepressants, exercise therapy, monotherapy, combination therapy, adjunctive therapy

## Abstract

Major depression disorder is most commonly treated with antidepressants. However, due to their side effects clinicians seek non-pharmacologic options, and one of these is exercise. The literature on the benefits of exercise for depression is extensive. Nevertheless, two recent reviews focusing on antidepressants vs. other therapies as a basis for clinical practice guidelines recommended mainly antidepressants, excluding exercise as a viable choice for treatment of depression. The aim of this perspective is to analyze the literature exploring the reasons for this discrepancy. Two categories of publications were examined: randomized controlled trials (RCTs) and meta-analyses or systematic reviews. Based on this reassessment, RCTs comparing exercise to antidepressants reported that exercise and antidepressants were equally effective. RCTs comparing exercise combined with antidepressants to antidepressants only reported a significant improvement in depression following exercise as an adjunctive treatment. Almost all the reviews examining exercise vs. other treatments of depression, including antidepressants, support the use of exercise in the treatment of depression, at least as an adjunctive therapy. The two reviews examining pharmacologic vs. non-pharmacologic therapies as a basis for clinical practice guidelines examined limited evidence on exercise vs. antidepressants. In addition, it is possible that academics and health care practitioners are skeptical of viewing exercise as medicine. Maybe, there is a reluctance to accept that changes in lifestyle as opposed to pharmacological treatment can alter biological mechanisms. Longitudinal studies are needed for assessing the effectiveness of exercise in real clinical settings, as well as studies exploring dose-response relationship between exercise and depression.

## Introduction

The perception of exercise as medicine has been discussed in relation to health conditions such as cognitive decline (e.g., Nagamatsu et al., 2014), cancer (e.g., Lin et al., 2016), cardiac rehabilitation (e.g., Almodhy et al., 2016), schizophrenia (e.g., Firth et al., 2015), alcohol use disorders (e.g., Hallgren et al., 2017), and all-cause mortality (e.g., Eklund et al., 2016). One meta-epidemiological study on mortality outcomes concluded that in a number of health conditions, such as heart failure, stroke, and diabetes, exercise and various pharmacological treatments are similar in their potential to extend longevity (Naci and Ioannidis, 2013). Thus, exercise interventions should be considered as a viable alternative to, or combination with, drug therapy (Naci and Ioannidis, 2013). It is therefore not surprising that comparative assessments of exercise and drug treatments were performed for some conditions, such as sleep disorders (Buman and King, 2010), chronic pain (Ambrose and Golightly, 2015), and mental health (Firth et al., 2016).

Probably more than for other health conditions, comparative benefits of exercise and pharmacologic treatments have been examined and discussed in relation to depression (e.g., Stubbs et al., 2015; Gartlehner et al., 2016). According to the WHO (World Health Organization), depression is the leading cause of disability worldwide (WHO, 2016 http://www.who.int/mediacentre/factsheets/fs369/en/).

Major depression disorder (MDD) is most commonly treated with antidepressant medication, where second-generation antidepressants are the most commonly prescribed drugs (Agency for Healthcare Research and Quality [AHRQ], 2016). However, many patients do not respond to antidepressant medications, or experience side effects (Gartlehner et al., 2016). In addition, increasing evidence indicated a large placebo response, making it more challenging for novel medications to demonstrate their effectiveness (Rutherford et al., 2012). Therefore, clinicians and patients seek non-pharmacologic options for treating depression – and one of them is physical exercise (e.g., Martiny et al., 2012).

The literature on the benefits of exercise for both minor and major depressive symptoms is extensive, with exceptionally numerous reviews perhaps outnumbering randomized controlled trials (RCTs) (e.g., Mead et al., 2009; Rethorst et al., 2009; Krogh et al., 2011; Rimer et al., 2012; Robertson et al., 2012; Cooney et al., 2013; Danielsson et al., 2013; Silveira et al., 2013; Josefsson et al., 2014; Mura et al., 2014; Knapen et al., 2015; Nyström et al., 2015; Stubbs et al., 2015; Kvam et al., 2016; Schuch et al., 2016b). In addition to exercise, additional studies (Adamson et al., 2016) and reviews (Zhai et al., 2015; Hallgren et al., 2016; Liu et al., 2016; Schuch et al., 2017) in recent years examined the relationship between sedentary behavior, physical activity, and depression. Interestingly, a recent study has shown that a 1-week of forced sedentary behavior may cause bad mood or depression in active individuals (Edwards and Loprinzi, 2016). Furthermore, it has been found that people with depression are at increased risk of sedentary behavior (Dugan et al., 2015; Schuch et al., 2017), which may cause cardiovascular diseases and metabolic syndromes (Gardner-Sood et al., 2015).

Along with RCTs and reviews examining exercise as a treatment for depression, there have been attempts to explore the mediating biological mechanisms explaining the reduction in depression in MDD as a result of exercise (Kandola et al., 2016; Schuch et al., 2016a). One explanation is hippocampus plasticity (Kandola et al., 2016). It has been shown that the hippocampus in depressed individuals may be affected by neuron atrophy (Mendez-David et al., 2013). Aerobic exercise has the potential to promote neuroplasticity and thus facilitate the function of the hippocampus (Erickson et al., 2011). Through increasing neuroplasticity in the hippocampus, it may be possible to generate structural changes that affect the region’s functioning and contribute to the alleviation of cognitive malfunction in MDD (Kandola et al., 2016). It has also been hypothesized that there is a relationship between the decline in neurogenesis and depressed mood (Duman et al., 1997). Based on the above, it was concluded that the anti-depressive effects of exercise are due to physiological changes that result in hippocampal neurogenesis (Ernst et al., 2006).

One mechanisms by which exercise could potentially facilitate this neurogenesis is the brain-derived neurotrophic factor (BDNF). A growing number of studies, performed both on animal models of depression and on depressed humans, have focused on the neurotrophic hypothesis of depression (Neto et al., 2011). According to this hypothesis, several alterations in the levels of neurotrophins, particularly of the BDNF, might produce the structural and neurochemical changes that underlie depression (Neto et al., 2011). Both pharmacological and non-pharmacological interventions for depression have been shown to produce changes in the levels of neurotrophins. BDNF increases have been reported to follow the administration of antidepressant drugs (Czubak et al., 2009), which suggests that BDNF expression may mediate the action of antidepressants. Furthermore, when exercise is combined with antidepressants, BDNF levels were found to increase in as little as two days, compared with two weeks with antidepressants alone (Russo-Neustadt et al., 2001).

Another mechanism for enhancing neurogenesis is serotonin. Adaptations in the serotonergic system may serve as potential facilitators of the antidepressant effects of exercise (Schuch et al., 2016a). As a result, antidepressant medications available today target the release and reuptake of serotonin. Exercise increases tryptophan hydroxylase (Chaouloff et al., 1989), which is necessary for serotonin synthesis. Results of animal studies point to a relationship between serotonin elevation and neurogenesis (Brezun and Daszuta, 2000).

According to Schuch et al. (2016a), it is possible that the antidepressant effect of exercise is caused by the interaction of several neurobiological mechanisms rather than by one mechanism exclusively. It is certain that exercise generates both acute and chronic responses, mainly in hormones, neurotrophines, and inflammation biomarkers (Schuch et al., 2016a).

It is, therefore, not surprising that quite a few attempts have been made to compare the effects of exercise to other treatments, including drug treatments, in various depressive disorders, specifically MDD. Four reviews on this topic were published in 2016. Two meta-analyses examining the efficacy of exercise as a treatment for major depression concluded that exercise as a treatment for depression can be recommended as a stand-alone treatment or as an adjunct to antidepressant medication (Kvam et al., 2016), and that exercise can be considered an evidence-based treatment for the management of depression (Schuch et al., 2016b). On the other hand, two systematic reviews comparing antidepressants to other therapies – *including exercise* – as a basis for clinical practice guidelines for depression, disregarded exercise in their recommendations. One concluded that “The American College of Physicians recommends that clinicians select between either cognitive behavioral therapy or second-generation antidepressants to treat patients with major depressive disorder…” (Qaseem et al., 2016, p. 355), and the other that “given comparable efficacy, cognitive behavioral therapy and antidepressants are both viable choices for initial MDD treatment” (Gartlehner et al., 2016, p. 338). The aim of this perspective is to analyze the available literature on the efficacy of exercise vs. antidepressants in the treatment of depression and to suggest a few explanations for this discrepancy.

### Publications Examined

Two categories of publications were examined: RCTs (**Table 1**) and meta-analyses or systematic reviews (**Table 2**).

**Table 1:** Randomized controlled trials (RCTs) comparing exercise to antidepressants in the treatment of depression.

Study	Participants	Treatment groups	Exercise	Duration	Conclusion
**Exercise vs. antidepressants (monotherapy comparisons)**					
Blumenthal et al., 1999	MDD Older adults,	1.Group exercise 2 Antidepressants 3.Combined	Three times/week Walking or jogging	4 months	Exercise and antidepressants equally effective
Blumenthal et al., 2007	MDD Adults,	1. Group exercise 2. Home-based exercise 3. Antidepressants 4. Placebo pills	Three times/week Walking or jogging	4 months	Participants in either exercise or antidepressants groups tended to show greater improvement in comparison with placebo participants
Hoffman et al., 2011 (follow-up of Blumenthal et al., 2007)	MDD Adults			1 year follow-up	No differences between treatment groups. Those who reported regular exercise following the intervention - the least likely to be depressed at follow-up
Brenes et al., 2007	Minor depression Older adults	1.Group exercise 2. Antidepressants 3.Usual care (discussions on health status)	Three times/week Aerobic and resistance	4 months	Both antidepressants and exercise led to improvements as compared to the usual care. Individuals in the exercise condition also improved in physical functioning

**Exercise combined with antidepressants vs. antidepressants only (combination comparisons)**					
Knubben et al., 2007	MDD Adults, inpatients	1. Aerobic exercise + antidepressants 2. Low-intensity + antidepressants	1. Individually treadmill walking 2. Individually stretching and relaxation	10 days Every day	Aerobic exercise as add-on therapy significantly improved depression. The proportion of patients with a clinical response was larger for the aerobic exercise group
Pilu et al., 2007	MDD Treatment-resistant women	1. Physiological strengthening + antidepressants 2. Antidepressants	Group cardio-fitness machines – aerobics and strengthening Two times/week	8 months	Exercise group showed a significant depression improvement
Mota-Pereira et al., 2011	MDD Treatment-resistant adults	1. Aerobic exercise + antidepressants. 2. Antidepressants only	Home-based, five times/week (1 day/week supervised	12 weeks	In exercise group, 21% showed response and 26% remission. None in control showed response or remission
Schuch et al., 2011	MDD Adults inpatients	1. Aerobic exercise + antidepressants. 2. Antidepressants only	Stationary bike, or treadmill or an elliptic, on individual basis, Three times/week	Through-out hospitalization	At 2 weeks, – both groups achieved improvements in depressive symptoms and quality of life, but difference favorable to exercise group at discharge.
Chalder et al., 2012	MDD Adults	1. Facilitated physical activity + usual care (58% antidepressants) 2. Usual care (56% antidepressants)	Three face to face sessions and 10 telephone calls with a trained physical activity facilitator	8 months.	Facilitated physical activity did not improve depression or reduce use of antidepressants compared with usual care alone, after 4, 8, and 12 months
Schuch et al., 2015	MDD Adults inpatients	1. Aerobic exercise + antidepressants. 2. Antidepressants only	Treadmill or bike or transport machine, on individual basis, Three times/week	Through-out hospitalization	Exercise group improved significantly more than control group on depressive symptoms and quality of life, as noticed at the second week of hospitalization and at discharge
Carneiro et al., 2015	MDD Adult women	1. Aerobic exercise + antidepressants 2. Antidepressants only	Traditional games, natural circuit workouts with resistance bands, jump ropes, fitness balls, brisk walking, and dancing, Three times/week	4 months	Exercise group decreased in depression, in anxiety and in stress and improved in physical functioning as compared to the control group
Kerling et al., 2015	MDD Adults inpatients	1. Aerobic exercise + CBT + antidepressants (only 77% antidepressants) 2. Usual care – CBT + antidepressants (only 75% antidepressants)	Bicycle ergometer followed by personal preference for cross trainer, stepper, arm ergometry, treadmill, recumbent, or a rowing ergometry Three times/week	6 weeks	Decline in depressive symptoms in both groups. Significantly more in exercise group classified as responders - at least 50% reduction in depression. Exercise group improved in physiological measures
Murri et al., 2015	MDD Older adults	1. High-intensity aerobic exercise + antidepressants 2. Low-intensity aerobic exercise+ antidepressants 3. Antidepressants only	1. High-intensity, progressive, mainly bicycles 2. Low-intensity, non-progressive mainly bicycles. Three times/week	24 weeks	Remission occurred in 81% of high-intensity 73% of low-intensity 45% of antidepressants only
Legrand and Neff, 2016	MDD Adult inpatients	1. Aerobic exercise + antidepressants 2. Placebo exercise + antidepressants 3. Antidepressants only	1. Walking or running mostly on individual basis 2. Stretching	10 days upon hospitalization, Every day	Both aerobic and stretching improved. A larger effect size in aerobic exercise. No change in depressive symptoms in control group
Mather et al., 2002	Minor depression, Older adults poorly responsive to depressive symptoms	1. Exercise + antidepressants 2. Health education + antidepressants	Endurance, strength and stretching Two times/week	10 weeks	Significant higher proportion - 55% – of exercise group than control – 33% experienced a greater than 30% decline in depression

**Table 2:** A map of RCTs in reviews comparing exercise to antidepressants in the treatment of depression, and the conclusions regarding the effect size of exercise.

Reviews	Reviews comparing the effect size of exercise vs. antidepressants in a specific sub-analysis			Reviews assessing a general effect size of exercise	Conclusion
	RCTs included in monotherapy comparison	RCTs included in combination comparison	RCTs together in mono and combination	All RCTs comparing exercise to other treatments including antidepressants	
Rethorst et al., 2009 Meta-analysis of exercise vs. other treatments	2 unpublished papers, 1 irrelevant				A significant overall effect size of exercise. ***No difference between effect sizes of exercise vs. antidepressants,*** but insufficient suitable studies. Supports the use of exercise in the treatment of major depression.
Mead et al., 2009 Cochrane review of exercise vs. other treatments			Blumenthal et al., 1999, 2007		Generally, exercise reduced depression. ***No difference between effect sizes of exercise vs. antidepressants.*** It is reasonable to recommend exercise to people with depressive symptoms but no accurate information
Krogh et al., 2011 Meta-analysis and systematic review of exercise vs. other treatments				Mather et al., 2002; Blumenthal et al., 2007	The ***general effect of exercise on depression is short-term***, little evidence of a long term beneficial effect. High quality trials, with long term follow-up, are required
Robertson et al., 2012 Meta-analysis and systematic review of exercise vs. other treatments				Knubben et al., 2007; Mota-Pereira et al., 2011	***A large (general) effect size of exercise*** (walking) on depression
Cooney et al., 2013 Cochrane review of exercise vs. other treatments			Blumenthal et al., 1999, 2007; Brenes et al., 2007		***No difference between exercise and antidepressants***. Exercise may be as effective as antidepressants, but small number of trials and participants
Silveira et al., 2013 Meta-analysis and systematic review of exercise vs. other treatments (some non-RCTs included)				Blumenthal et al., 2007; Knubben et al., 2007	Exercise is an efficient alternative treatment for depression (***general effect size*)**, specifically in old age and for mild depression. Based on Blumenthal et al. (1999, 2007) studies, aerobic training is as effective as antidepressants
Knapen et al., 2015 (review of reviews of exercise vs. other treatments				Cooney et al., 2013; Silveira et al., 2013	***General effect size***: For mild to moderate depression – exercise comparable to antidepressants, for severe depression – exercise valuable as complementary therapy
Mura et al., 2014 Meta-analysis and systematic review (some non-RCTs included)		Blumenthal et al., 1999; Mather et al., 2002; Knubben et al., 2007; Pilu et al., 2007; Mota-Pereira et al., 2011; Schuch et al., 2011			***A strong effectiveness* of *exercise combined with antidepressants***, but the majority of studies suffered from methodological weaknesses.
Schuch et al., 2016b Meta-analysis of exercise vs. other treatments				Blumenthal et al., 2007; Brenes et al., 2007; Pilu et al., 2007; Mota-Pereira et al., 2011; Kerling et al., 2015; Schuch et al., 2015	***A large and significant effect size of exercise, larger for MDD, for aerobic exercise, and for supervised formats.*** Criticized previous meta-analyses for underestimating benefits of exercise due to publication bias. Not right to calculate exercise-drugs as exercise may potentially overlap with potential mechanisms of drugs.
Kvam et al., 2016 Meta-analysis of exercise vs. other treatments	Blumenthal et al., 1999, 2007	Pilu et al., 2007; Mota-Pereira et al., 2011; Schuch et al., 2011			***Monotherapy: Exercise efficient as drugs. Combination: Moderate effect size trending toward significance. Exercise can be recommended as a stand-alone treatment and as an adjunct to antidepressant medication***
Qaseem et al., 2016 Systematic review of all treatments	Blumenthal et al., 2007; Hoffman et al., 2008 (irrelevant as not assessing depression)	Blumenthal et al., 1999			***Exercise vs. antidepressants: no difference in remission in both mono and combination therapy. However, exercise not recommended as a treatment of depression.*** Low quality evidence
Gartlehner et al., 2016 Systematic review of all treatments	Blumenthal et al., 2007	Blumenthal et al., 1999; Murri et al., 2015			***Exercise vs. antidepressants: no difference in monotherapy, improvement in combination therapy. However, exercise not recommended as a treatment of depression***. Low to moderate quality evidence

All RCTs published in 1999–2016 that are included in systematic and/or meta-analyses reviews published from 2009 to 2016 were examined, as well as two recent RCTs found in PubMed search. RCTs were excluded if they assessed participants with additional co-morbid diagnoses, such as cardiovascular diseases (e.g., Blumenthal et al., 2012), or if they assessed two kinds of interventions as add-on therapy – for example chronotherapy vs. exercise (Martiny et al., 2012). One group of RCTs compared exercise to antidepressants – monotherapy comparisons, and the other compared exercise combined with antidepressants to antidepressants only – combination comparisons (**Table 1**).

As a collection of RCTs does not reflect a general effect size, meta-analyses, Cochrane reviews and systematic reviews providing an effect size of exercise vs. antidepressants in the treatment of depression were examined. As a large number of meta-analyses and other reviews were conducted in the last decade, it was decided to screen only reviews from the last seven years (2009–2016). Interestingly, in spite of the large number of reviews, none of them focused solely on exercise vs. antidepressants. One group compared exercise to other treatments of depression, including antidepressants, and the other compared antidepressants to other therapies, including exercise. More specifically, the present review investigated: (1) whether comparisons were conducted specifically between exercise and antidepressants (as opposed to exercise vs. all other treatments together, or antidepressants vs. all other treatments together), (2) which RCTs comparing exercise to antidepressants were included in these reviews, (3) which conclusions were drawn from these comparisons, and (4) whether all published RCTs conducting such comparisons were included in the reviews.

### Summary and Conclusions of the Findings

#### Randomized Controlled Trials

##### Exercise vs. antidepressants – monotherapy comparisons (**Table 1**)

Three RCTs compared 4 months of exercise to antidepressants– two for MDD (Blumenthal et al., 1999, 2007) and one for minor depression (Brenes et al., 2007). Two were conducted on older adults (Blumenthal et al., 1999; Brenes et al., 2007). One study (Hoffman et al., 2011) was a follow-up to a previous study (Blumenthal et al., 2007). The Blumenthal et al. (1999, 2007) studies included aerobic exercise, and the Brenes study a combination of aerobic and resistance exercises.

Conclusion: **All these studies reported that exercise and standard antidepressant treatments were equally effective.**

##### Exercise combined with antidepressants vs. antidepressants only – combination comparisons (**Table 1**)

Eleven RCTs compared exercise as an adjunctive treatment to antidepressants (combination comparisons) – 10 for MDD and one for minor depression (**Table 1**). The duration of the exercise period varied from 10 days (Knubben et al., 2007; Legrand and Neff, 2016), to 6 weeks (Kerling et al., 2015), 10 weeks (Mather et al., 2002), 3 months (Mota-Pereira et al., 2011), 4 months (Carneiro et al., 2015), 6 months (Murri et al. (2015), 8 months (Pilu et al., 2007), 12 months (Chalder et al., 2012), to throughout a hospitalization period (undefined time period) (Schuch et al., 2011, 2015). Control groups included antidepressants only (Mather et al., 2002; Pilu et al., 2007; Mota-Pereira et al., 2011; Schuch et al., 2011, 2015; Chalder et al., 2012; Carneiro et al., 2015; Kerling et al., 2015), light exercise with both exercise groups receiving antidepressants (Knubben et al., 2007; Legrand and Neff, 2016), and antidepressants only (Murri et al., 2015). The exercise mode included mostly aerobics (Knubben et al., 2007; Mota-Pereira et al., 2011; Schuch et al., 2011, 2015; Kerling et al., 2015; Murri et al., 2015; Legrand and Neff, 2016) or aerobic and strength (Pilu et al., 2007); aerobic, strength, and stretching exercises (Mather et al., 2002**)**; aerobics and strength exercises, games and dancing (Carneiro et al., 2015), and facilitated physical activity chosen and performed individually by participants (Chalder et al., 2012). The studies using exercise in a control group used light stretching (Knubben et al., 2007; Legrand and Neff, 2016) and low-intensity aerobics (Murri et al., 2015). Most studies assessed adults in general; only two studies investigated older adults (Mather et al., 2002; Murri et al., 2015).

Of special interest are the studies using exercise placebo groups as a control group, in which improvements were observed in the aerobic exercise as compared to stretching (Knubben et al., 2007; Legrand and Neff, 2016), and the Murri et al. (2015) study that showed the greatest improvement in high-intensity aerobics, followed by low intensity aerobics, followed by antidepressants only. The Chalder et al. (2012) study only gave guidance about exercise but did not provide an exercise program.

Conclusion: All studies but one (Chalder et al., 2012) informed that patients using exercise as an adjunctive treatment for depression showed a significant depressive improvement after the exercise period, and/or that the proportion of patients with a clinical response was larger for the exercise group than the control.

### Meta-Analyses or Systematic Reviews

**Table 2** presents a map of RCTs comparing exercise to antidepressants in the meta-analyses or systematic reviews.

**Almost all reviews examining exercise vs. other treatments of depression, including antidepressants, support the use of exercise in the treatment of depression, at least as an add-on therapy.** Earlier reviews, which included only a few RCTs, were more careful in actually recommending exercise. For example, one review stated that “it is reasonable to recommend exercise…” (Mead et al., 2009, p. 14). Another review pointed out that “… exercise may be as effective as psychological or pharmacological treatments…” (Cooney et al., 2013, p. 35). Later reviews were more conclusive, claiming “a strong effectiveness of exercise combined with antidepressants” (Mura et al., 2014, p. 503); “Overall, our results provide robust evidence that exercise can be considered an evidence-based treatment for the management of depression.” (Schuch et al., 2016b, p. 47); and “Physical exercise is an effective intervention for depression. It also could be a viable adjunct treatment in combination with antidepressants” (Kvam et al., 2016, p. 67).

**On the other hand, the two recent reviews from 2016 assessing antidepressants vs. other treatments of depression, including exercise, did not recommend exercise for the treatment of depression** (Gartlehner et al., 2016; Qaseem et al., 2016). However, when comparing exercise to antidepressants, these reviews examined mainly the Blumenthal et al. (1999, 2007) studies, excluding other RCTs comparing exercise to antidepressants that were included in other recent reviews.

## Discussion

Exercise vs. pharmacologic treatment of depression in the clinical practice guideline of the American College of Physicians – is it evidence-based?

It appears that the reviews examining pharmacologic vs. non-pharmacologic treatments of depression as a basis for clinical practice guidelines examined limited evidence on exercise vs. antidepressants, and thus disregarded exercise as a viable choice for treating depression as a stand-alone treatment or as an add-on therapy. This position is contrary to the reviews examining exercise vs. other treatments for depression, including antidepressants, which generally recommend exercise as a stand-alone and/or as adjunctive treatment for depression. The evidence is even greater when considering two additional recent well-designed RCTs not included in any of the reviews (possibly because they were published later than the RCTs mentioned in the reviews) which pointed out the effect of exercise as a complement to antidepressant medication (Carneiro et al., 2015; Legrand and Neff, 2016) (**Table 1**). Furthermore, while the underlying biological mechanisms mediating between exercise and reduced depressive symptoms are not entirely clear, it is apparent that exercise induces both acute and chronic responses, particularly in hormones, neurotrophines, and inflammation biomarkers, and that there is an association between hippocampus neurogenesis as a result of exercise and depressive symptoms’ improvement (Schuch et al., 2016a).

Is exercise medicine for the treatment of depression?

Based on the present review, which examined most or all RCTs published in 1999–2016, and most or all meta-analyses/systematic reviews published in 2009–2016, it can be stated that exercise is an evidenced-based medicine for depression – at least as an add-on to antidepressants. Furthermore, people with depression are at increased risk of sedentary behavior (Dugan et al., 2015; Schuch et al., 2017), which may cause cardiovascular diseases and metabolic syndrome (Gardner-Sood et al., 2015). Thus, exercise contributes to the physical health in addition to mental health. It is also worth mentioning the adverse effects commonly associated with drugs, including constipation, diarrhea, dizziness, headache, insomnia, nausea, adverse sexual events, and somnolence (Qaseem et al., 2016), which may further support the use of exercise as a viable alternative or adjunctive pharmacotherapy.

It is unclear why exercise was disregarded as a viable choice for treating depression in the clinical practice guidelines recommended in the two recent reviews (Gartlehner et al., 2016; Qaseem et al., 2016). Is there a reluctance among academics and health care practitioners to view exercise as medicine? Do they caution that there is no strong evidence to suggest that modifiable lifestyle factors as opposed to pharmacological treatment can alter biological mechanisms in similar pathways or similar dynamics to biochemical interventions?

Interestingly, this argument was raised by Nagamatsu et al. (2014) regarding the effect of exercise on the brain and cognition in old age. These authors made the case that despite the large and consistent pool of evidence generated over the past five decades linking exercise to improved cognitive functions in older adults, skepticism remains and health practitioners continue to hinder the adoption of exercise as a legitimate medical strategy for the prevention of cognitive decline.

Future directions of research should include dose-response interventions to determine the precise dose of exercise required to maximize the benefits for depression. In addition, more studies are needed to inquire the underlying molecular and cellular mechanisms mediating between exercise and depression. Furthermore, another important issue for assessing the benefits of exercise for depression is its effectiveness as opposed to efficacy (Beedie et al., 2016). While efficacy refers to the ability of exercise to achieve the desired effect under well controlled circumstances, effectiveness refers to the ability of exercise to affect depression in real life situations. Therefore, longitudinal observational studies exploring the benefits of exercise in depression are needed, which assess adherence issues as well as economic and professional matters.

## Author Contributions

The author confirms being the sole contributor of this work and approved it for publication.

## Conflict of Interest Statement

The author declares that the research was conducted in the absence of any commercial or financial relationships that could be construed as a potential conflict of interest.
